# Comparative cariostatic efficacy of a novel Nano-silver fluoride varnish with 38% silver diamine fluoride varnish a double-blind randomized clinical trial

**DOI:** 10.4317/jced.54995

**Published:** 2019-02-01

**Authors:** Sunnypriyatham Tirupathi, Nirmala SVSG, Srinitya Rajasekhar, Sivakumar Nuvvula

**Affiliations:** 1 MDS, Assistant Professor, Department of Pediatric Dentistry, Mallareddy Institute of Dental Sciences, Hyderabad, India; 2 MDS, Professor, Department of Pediatric Dentistry, Narayana dental college and hospital, Nellore, India; 3 MDS, Private practitioner, Pediatric Dentistry, India; 4 MDS, Head of department, Department of Pediatric Dentistry, Narayana Dental College and Hospital, Nellore, India

## Abstract

**Background:**

To evaluate the clinical cariostatic efficacy of a concocted 5% Nano-silver incorporated Sodium fluoride (NSSF) dental varnish with 38% Silver diamine fluoride (SDF) in preventing the progression of dentinal caries of primary molars.

**Material and Methods:**

A total of 159 active dentinal carious lesions in primary molars (from 50 children) were selected and randomly divided into two groups; NSSF and SDF. Varnish application was performed at the baseline visit and follow-up is carried out at 1, 3, 6 and 12 months. Parameters such as caries activity, depth, size, colour, and presence or absence of pain were noted at baseline and follow-up visits.

**Results:**

The intergroup comparison of caries activity status did not reveal significant difference between the number of active and arrested caries lesions in NSSF and SDF groups at any visit (*p* >0.05) during the 12-month follow-up. There was no difference between overall failure and success rates between the two groups at any follow-up period (*p*-0.48).

**Conclusions:**

Annual application of 5% NSSF is better than or equal to 38% SDF in preventing the progression of dentinal caries of primary molars. NSSF does not cause dark staining of dentinal tissue compared to the SDF and the use of NSSF can be recommended in children.

** Key words:**Children, Nano-silver fluoride, Primary teeth, Silver diamine fluoride.

## Introduction

Despite the advances in dental care, dental caries remains one of the most common oral disease affecting children. Traditional approaches of treating dental caries include surgical removal of the diseased dental tissue, followed by the placement of suitable restorative material. Traditional approaches are replaced by minimal invasive approaches to arrest the progression of caries. Variety of fluoride based chemotherapeutic agents were developed and tested for their preventive and arresting effect on dental caries. Every agent has its merits and demerits and varied efficacy has been reported with different agents (1).

Caries arresting treatments can be provided at the community level to arrest the progression of dental caries at regular intervals. A metal ion based topical fluoride preparation named Silver diamine fluoride (SDF) has been drawing increased attention contemporarily, due to its efficacy in arresting the progression of dental caries. Asian countries such as Japan and China pioneered its use, however due to the promising results and availability of clinical trials it has been popularised throughout the world. The most commonly used concentration of SDF is 38% (44,800 ppm F), nevertheless, 30% (35,400 ppm) and 12 % (14,150 ppm) were also used to arrest caries progression (2). The clinical efficacy of 38% SDF in arresting progression of dentinal caries was found to be around 65.9% (3). The major drawback of SDF is, staining of carious tissue to dark black due to the oxidation process of ionic silver contained in its formulation, along with ulceration and staining of oral tissues which are painful. However, soft tissue stains formed due to SDF is usually reversible (4).

Nano-silver (AgNPs) is one of the potent antimicrobial agents and its efficacy against cariogenic bacteria such as *S.mutans* is established *in-vitro*. Antibacterial activity of AgNPs is 25 fold higher than chlorhexidine (5,6), however, Nano silver has an additional anti-viral and anti-fungal efficacy. Haghgoo and co-workers suggested the incorporation of Nano silver in dental varnishes (7). Clinical studies report that, Nano-silver containing fluoride varnish preparation showed better efficacy than the control to arrest caries progression in primary teeth (8). In an in-vitro comparison of antimicrobial efficacy and cytotoxicity between SDF and nano silver based fluoride varnish preparation carried out by Targino and co-workers, the antimicrobial efficacy of Nano silver based fluoride varnish preparation was better than SDF, however the cytotoxity of Nano silver based fluoride varnish preparation was less when compared to silver diamine fluoride (9).

To the best of our knowledge no studies related have been reported till date, comparing the clinical efficacy of SDF and Nano-silver based fluoride varnish preparation in arresting carious lesion progression *in-vivo*. Hence this study was undertaken to evaluate and compare the clinical cariostatic efficacy of 5% NSSF to that of 38% SDF.

## Material and Methods

CONSORT guidelines were followed in designing, interpreting and analysing this study. This was a community based, single-centre, randomized (balanced randomization of 1:1), double-blinded, active-controlled parallel-group study. Screening was carried out in a government primary school. Children aged between 6- 10 years with active dentinal caries and who met the inclusion and exclusion criteria were recruited.

This current study has been carried out in a community setting at a government primary school adopted by the Department of Pedodontics and Preventive Dentistry, for a study period of 12 months from February 2015 to February 2016 after clearance from the Institutional ethical committee and the university (Registration number D148407051) was obtained. Trail was also registered under Clinical trial registry India (CTRI/2017/03/008135).

-Inclusion criteria: Children aged 6 to 10 years with active dentinal caries in primary molars; absence of pulp involvement, pain, mobility, abscess, sinus, fistula; participants should be free of any systemic diseases; written informed consent from the parents/guardians.

-Exclusion criteria: Children whose parents refused to give consent: primary teeth with irreversible pulpitis; tooth close to exfoliation.

One of the two dental varnishes compared in this study was commercially available Saforide® (38% Silver diamine fluoride J. Morita; Toyo Seiyaku Kasei Ltd. Japan) and the other was manually prepared 5% NSSF.

-Preparation of 5% Nano silver incorporated sodium fluoride.

Weight dilution method described by Haghoo *et al.* 2014 was adopted in the preparation of 5% NSSF (7). Silver nano-particle powder 0.5 grams (99.5% pure; Particle size of less than 100 nanometre containing PVP (polyvinyl pyridoline) as a dispersant i.e., water soluble; Sigma Aldrich) (LOT Number- MKBQ8287V) was added to 10 ml of 22,600 ppm of slow release Sodium fluoride varnish (FLUORITOP™-SR) in a light proof brown bottle and vigorous stirring is performed to achieve uniform dispersion of Nano-silver particles.

Primary outcome of interest is clinical cariostatic efficacy of 5% NSSF to that of 38% SDF in preventing progression of dentinal caries in primary molars.

-Sample size and selection.

Each tooth with dentinal carious lesion was considered single unit. The sample size was estimated to be a minimum of 50 lesions for each varnish group. Extra sample size of 30% was taken in both the groups to compensate for the possible follow up loss of any reason.

Sample size calculation was based on a previous study by Santos and co-workers (8).A total of 240 children were screened for the eligibility and 50 were included as they met the inclusion criteria. A total of 159 lesions in 50 children were divided randomly into blocks of four lesions(Block randomisation). These blocks were given specific numbers and were divided into two groups based on computer generated random numbers (https://www.randomizer.org/). Group A (5% NSSF)-76 lesions: Group B (38% SDF)-83 lesions. To achieve allocation concealment, randomisation was performed by an examiner who was not involved in the study. The children in this study were blind to the type of treatment and outcome evaluation was done by an examiner who was blind to the type of intervention.

-Intervention.

After thorough oral prophylaxis, baseline ‘dmft’ was recorded. No effort was made to remove the caries or unsupported enamel for caries treatment in both the groups under cotton roll isolation. Initial cleaning of cavity was performed by using small cotton pellet followed by the application of single drop (0.1 mL) of either 5% NSSF or 38% SDF (Saforide; Toyo Seiyaku Kasei Co.Ltd., Osaka, Japan) with a disposable micro-applicator tip for 10 seconds and the cavity was closed with a cotton pellet for ten minutes and the child was instructed not to drink water or eat food for at least 45 minutes. One micro applicator tip was used for each lesion and discarded after single use. Both the treatments were performed with single application and no repetition. SDF was used as positive control group for evaluating cariostatic efficacy, as SDF is already established cariostatic agent for arresting carious lesion progression and how good a newer agent is comparatively, can be assessed.(3)

-Follow-up evaluation.

The teeth were assessed clinically using visual and tactile inspection by a trained blind examiner after 1 month, 3 months and 6 months and 12 months. Size of the carious lesion was classified as mild, moderate and enlarged based on Mount and Hume classification of caries (10). Depth of the carious lesion was measured using UNC-15 probe. Presence or absence of pain in the follow up visit was recorded using verbal questionnaire. Presence or absence of active caries was determined in both the groups based on Miller’s criteria followed in previous studies (8,11). According to Miller’s criteria, caries status was considered active if a blunt UNC-15 probe penetrated carious lesion easily, whereas, arrested caries was noted when blunt UNC-15 probe could not penetrate the dentine. Failures in the respective groups were treated by performing pulpectomy followed by full coverage restoration.

-Blinding.

Two undergraduate examiners who are blind to type of varnish used were involved in the application of two varnishes. These two examiners are also blind to colour change associated with each varnish. Evaluation of carious lesion after initial varnish application at baseline and at each follow up visit were conducted by two different postgraduate examiners who were blind to type of varnish used.

-Statistical analysis.

Data was recorded in excel sheets and statistical analysis was performed using SPSS for Windows release 19.0 (SPSS Inc., Chicago, IL, USA). Descriptive statistical methods to evaluate caries lesion Progression, whereas quantitative statistical methods to evaluate the depth of lesion were used. Cohen kappa for inter examiner reliability gave a total score of 0.9. Qualitative data was assessed and converted to quantitative data based on the number of failures and success in each group. Intergroup and Intragroup comparisons were made using Chi-square test. Intergroup comparison of the depth was done at each follow up visit using one-way ANOVA and with 95% confidence interval tukey post-hoc test was performed.

## Results

At the end of one year follow-up, there was a total follow-up loss of 12 (7.5% drop out percentage) of 159 lesions recruited at baseline. The reason for drop-outs being discontinuation of children and the drop-outs were excluded for the final data analysis ( Table 1).

**Table 1:**
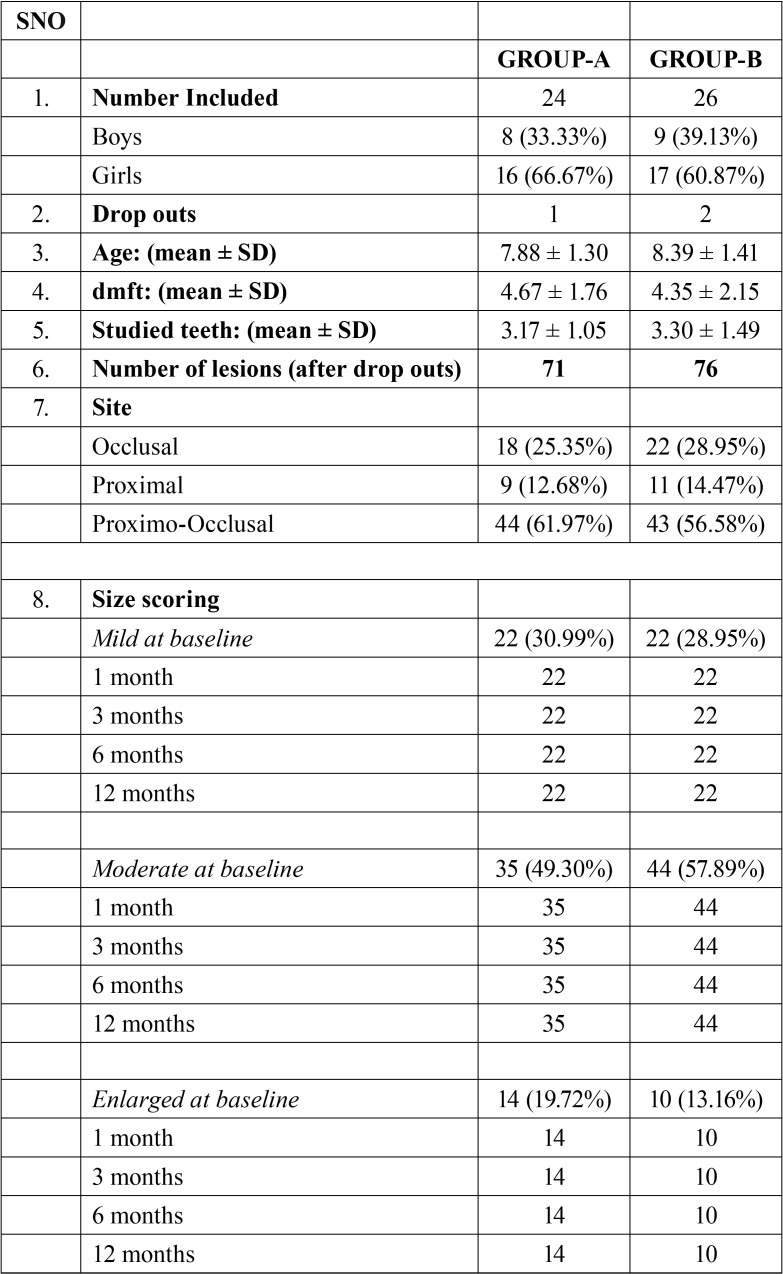
Table showing site and size and demographic variables.

Chi-square test revealed that, there was no statistically significant difference between the groups with respect to age, gender and ‘dmft’ at baseline. (*p*-value > 0.05).

Site: Out of 71 carious primary molars included in the NSSF group, 44 (61.97%) were Proximo-occlusal; 18 (25.35%) occlusal and 9 (12.68%) proximal. In the SDF group, out of 76 carious primary molars, 43 (56.58%) were proximo-occlusal, 22 (28.95%) occlusal and 11 (14.47%) proximal ( Table 1).

-Size: At the baseline visit, carious primary molars included for the study were classified basing on their size, as mild, moderate, enlarged in both the groups and recorded at each follow up visit. In the NSSF group, 22 (30.99%) were mild and 35 (49.30%) were moderate, whereas, 14 (19.72%) were enlarged. In the SDF group, 22 (28.95%) were mild and 44 (57.89%) were moderate while 10 (13.16%) were enlarged. In all the follow up visits at 1 month, 3 months, 6 months and 12 months, there was no drastic change in the size scoring of the lesion in both the groups ( Table 1).

-Depth: The depth of the carious lesions was measured at each visit and compared with the baseline in both the groups, intra and intergroup comparisons were made. In NSSF group, the mean depth at baseline was 2.94 ± 1.10 which did not change from baseline to one and 3 month’s follow up. However, there was an increase in the mean depth in NSSF group at 6 (3.12 ± 1.21) and 12 months (3.15 ± 1.23) follow up, which was highly significant statistically (*p*-value< 0.0001) ( Table 2). In the SDF group the baseline mean depth was 3 ± 0.74, which did not change at 1 month follow up visit, there was increase in the mean depth (3.02 ± 0.78) at three month follow up visit, however the difference from baseline mean depth was not statistically significant (*p*-value-0.079). There was increase in the mean depth at 6 months (3.25 ± 0.85) and 12 months (3.25 ± 0.85) follow up and the difference was found to be highly significant statistically (*p*-value < 0.0001) ( Table 2). Inter group depth comparison between NSSF and SDF group revealed that, there was no statistically significant difference in the mean increase in depth for both the groups at any follow up visit (*p*>0.05).

**Table 2:**
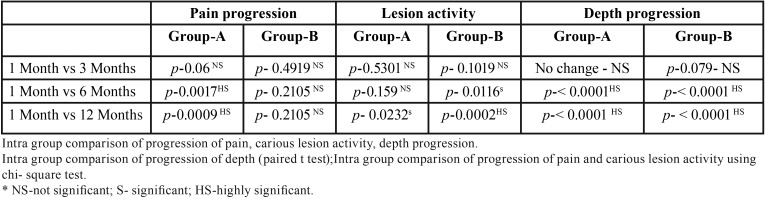
Intragroup Pain progression and carious lesion activity and depth progression.

-Pain: Presence or absence of pain was noted at each visit in both the groups and compared. There was no statistically significant difference between the number of children reporting pain or its absence in both the groups at each visit (*p*>0.05). Intragroup comparison of presence of pain revealed that, there was no statistically significant increase in the number of children reporting pain in NSSF group between one and three-month follow-up visits ( Table 2). Significant increase in the number of children reporting pain was observed in NSSF group between one and six month follow up as well as one month and twelve month follow up visits (*p*<0.05). In the SDF group there was no statistically significant increase in the children reporting pain from one to three, six and twelve month follow up visits (*p*>0.05) ( Table 2).

-Caries lesion activity: Caries activity status was recorded whether it was active or arrested in both the groups at each follow up visit. Intergroup comparison of both the groups revealed that, there was no significant difference between the number of active and arrested caries lesions between NSSF and SDF groups at any follow up visit (*p*>0.05). Intragroup comparison of NSSF group revealed that, there was no significant increase in the active caries lesions between 1 month to three month follow up visit (*p*-0.53). Similarly, no significant increase in the number of active caries lesions was observed between 1 month to that of six month follow up visit. Significant increase in the number of active carious lesions occurred between 1 month and twelve month follow up visits (*p*-0.02). In the SDF group there was no significant increase in the number of active lesions from 1 month to three month follow up visits. However, number of active carious lesions increased statistically from 1 month to six-month follow up visit (*p*-0.01). There was a highly significant increase in the number of active lesions from 1 month to 12 month follow up visit (*p*-0.0002) ( Table 2).

**Figure 1:**
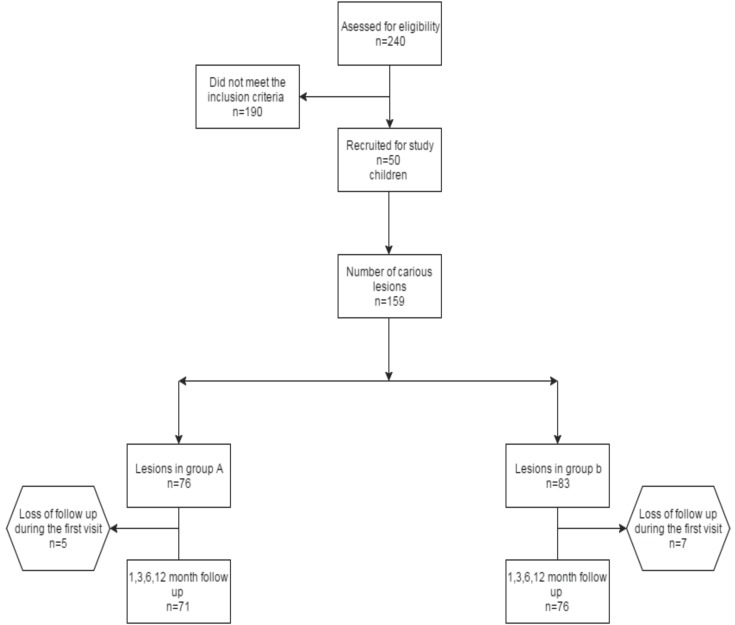
Flow chart showing number of lesions included, received intervention, lost during follow-up.

-Overall failures & success: Overall failures were assessed at each follow up visit, based on the criteria described previously i.e. presence of pain and/or presence of active carious lesion were considered as failure. Intra-group analysis revealed that, in both NSSF and SDF groups, significant overall failures occurred at the end of six and twelve months follow up visits. At the end of six months, there were 14 failures out of a total 71 in the NSSF group and 16 failures out of total 76 lesions in SDF group. However, the difference in the failures was not statistically significant (*p*-0.84). At the end of twelve month follow up, there were 16 failures out of a total 71 in NSSF group, whereas 22 failures out of total 76 lesions in SDF group, however, the difference is not statistically significant (*p*-0.48) ( Table 3, Table 4), (Fig. 1).

**Table 3:**
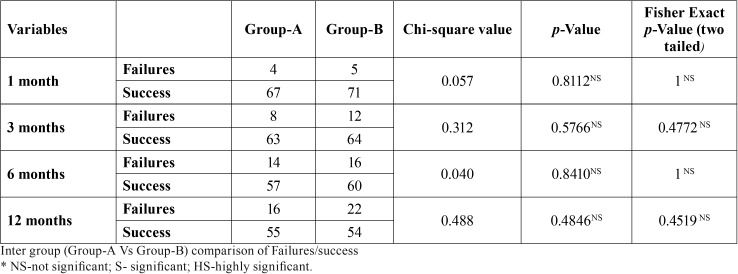
Overall failures & success- Intergroup.

**Table 4:**
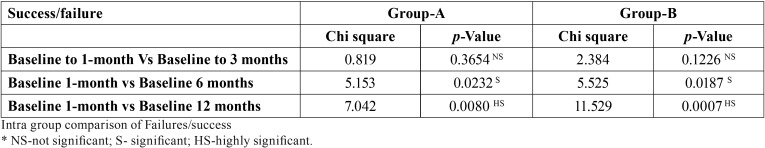
Overall failures & success- Intragroup.

## Discussion

The present study compared the clinical efficacy of annual application of 5% Nano-silver incorporated Sodium fluoride varnish (5% NSSF) with commercially available 38% SDF in preventing the progression of dentine caries lesion in primary molars. Preventive effects of both these agents were evaluated based on the number of failures in each group after specific follow up periods.

SDF showed excellent antibacterial action against cariogenic strains of *S.mutans* (12), and the proposed cariostatic effect of SDF is due to a combination of effects such as Silver salt stimulated sclerotic dentine formation and potent germicidal effect of Silver nitrates and the ability of fluoride to reduce the decay. Mei and co-workers reported that, the use of 38% SDF inhibited demineralization and preserved collagen from degradation in the demineralized dentin (13).

Application of SDF increases the micro-hardness of dentine in primary teeth as a result arrested dentine formed are more resistant to further caries attack (14). Numerous studies prove that SDF is efficient in arresting caries progression in both primary and permanent teeth (3,15-18).

Based on a systematic review by Marinho and co-workers, the caries preventive effect of plain Sodium fluoride varnish in primary teeth is reported to be around 37% and in permanent teeth is found to be 43% (1). Moreover, Gao and co-workers based on their systematic review reported that, SDF is better than plain 5% Sodium fluoride varnish in the prevention of dentinal carious lesions (3,18). To increase the antimicrobial activity of sodium fluoride varnish, the incorporation of other materials have been tried by few authors with positive results (19-22).

Nano silver based preparations are gaining popularity as excellent anti-bacterial, anti-fungal and anti-viral agents. The possible mechanisms of action of Nano-silver (23) are due to better contact with the microorganism. Nanometre scale Silver provides an extremely large surface area for contact with bacteria. The nanoparticles get attached to the cell membrane as well as penetrate inside the bacteria. Bacterial membranes contain sulphur-containing proteins and AgNPs, like Ag+, can interact with them as well as with phosphorus-containing compounds such as DNA, perhaps to inhibit the function. Silver nanoparticles can attack the respiratory chain in bacterial mitochondria and lead to cell death. AgNPs can have a sustained release of Ag+ once inside the bacterial cells, which may create free radicals and induce oxidative stress, further enhancing their bactericidal activity. Such interactions in the cell membrane would prevent DNA replications, which would lead to bacterial death.

At the concentrations used in dental materials, no toxicity and adverse effects of Nano-silver are reported so far. Cytotoxic studies reported that Nano-silver has lower cytotoxicity when compared to other dental materials (9,24). Nano-silver has the ability to inhibit plaque and biofilm formation by supressing growth of biofilm forming bacteria (25,26). Addition of nanosilver to dental composite resin had a significant effect on the reduction of the number of *S.mutans* and *L.bacillus* colonies (27-29). Incorporation of Nano-silver in the denture base acrylic resin polymer increased the anti-candidal effect (30) Antimicrobial efficacy of Nano-silver is inversely proportional to the size of Silver nano particles i.e., lesser the size, higher the antimicrobial efficacy (31). Nano silver based endodontic irrigants are also tested with good results (32).

Haghgoo and co-workers suggested the incorporation of Nano-silver in varnishes and reported, increased antimicrobial properties against cariogenic microorganisms such as *S.mutans* and *S.salivarius* (7). Randomized controlled clinical trials reporting the efficacy of Nano-silver containing fluoride varnishes over placebo are reported in the literature (8). Targino and co-workers conducted invitro antimicrobial study and reported that, Nano-silver containing fluoride preparation was equivalent to SDF and it has an additional advantage of low cytotoxicity than SDF (9).

In the present study, clinical cariostatic efficacy of once a year application of 5% NSSF preparation and 38% SDF in primary carious molar teeth were compared. Based on the results obtained after a twelve-month evaluation of both the groups, NSSF is equivalent to SDF in inhibiting dentine caries progression in primary teeth. Both the groups had almost equal number of failures at the end of the twelve month follow up and the difference was not statistically significant. The success rate of NSSF was 77%, when compared to the 71.05% success rate in SDF group. Overall success rate was slightly better in the NSSF group, compared to the SDF group, however the difference was not statistically significant.

Effectiveness of 5% NSSF might be due to synergism of its components (Nano silver and sodium fluoride). The effectiveness of the NSSF used in this study in arresting the progression of dentinal caries is slightly higher than other Nano-silver containing fluoride preparations previously reported in the literature (8). Santos and co-workers reported a success rate of 66.7% with their Nano silver fluoride preparation. In the present study, a success rate of 77% at the end of twelve-month follow up with NSSF was obtained. Furthermore, increased success rates in SDF group (71.05%) as opposed to 66.9% success in the study by Santos and co-workers was observed (8). Reasons for this increased success in the present study might be due to the following reasons.

1. In the present study, the children belong to optimally fluoridated area and also use fluoridated tooth paste.

2. Saforide® was used, which has a concentration of 38% SDF, whereas, Santos and co-workers used 30% SDF.

One interesting observation in the present study is; SDF caused dark staining when in contact with dentinal tissue. However, in few teeth this dark staining started to fade slowly at the start of 6 month follow up visit. Moreover, SDF has the capability to stain gingival tissues adjacent to the lesion and the stains of both hard and soft tissue was reversible.

At no point of time, adverse effects have been reported in both the groups. Hence, both NSSF and SDF are deemed safe to be used on children.

Cost-effectiveness: 5% NSSF is eight times economical compared to 38% SDF hence, its use at community level is cost effective and reliable.

-Limitations:

The limitations of this study could be less sample size as well as being conducted in a single-site. A multi-site study with larger sample size can be planned to generalise the results and confidently apply the results in clinical practice.

## Conclusions

Once annual application of 5% NSSF has same clinical efficacy as SDF in preventing the progression of dentinal caries in primary posterior teeth. The advantages of 5% NSSF over SDF is; it does not cause immediate dark staining of dentinal tissue, the reason being nano silver does not form oxides, no metallic taste, no painful ulceration and it is relatively economical, compared to SDF. Hence, the use of 5% NSSF can be recommended in children.

Both 5% NSSF and SDF can be considered as materials that can meet the WHO millennium goals as they are, simple, non-invasive, less technique sensitive and cost effective mode of treatment for caries arrest.
